# A New Method of Medicine

**Published:** 1886-03

**Authors:** 


### A NEW METHOD OF INTRODUCING MEDICINE INTO THE SYSTEM.

At a meeting of the French Academy of Medicine, held September 22, M. Brondel read a paper on the introduction of certain medicines into the system by means of electricity. If the electric current is made to pass through a solution of a salt, the salt is decomposed, the metallic base passing to the negative pole, and the acid, or metalloid, to the positive pole. The iodides are easily decomposed by electricity. In order to introduce iodine into the system, a rubber plate, moistened with a solution of iodide of potassium, is placed upon the surface of the body. Over this plate the negative pole of a battery is applied, while the positive pole is placed upon a part of the body toward which it is desired that the iodine travel. The iodine separates from the potassium, which remains at the negative pole, and passes with great rapidity through the tissues toward the positive pole. This may be demonstrated by testing with a starched paper, which becomes blue. A great number of substances can thus be made to traverse the tissues, and the applications of this discovery are numerous and important. M. Brondel has in this way cured uterine fibroids, a case of perimetritis, rheumatic ovarian, neuralgia, and several cases of chronic rheumatism.—*Le Progres Medical.*

### VINEGAR IN DIARRHŒA AND DYSENTERY.

Dr. Amos Sawyer, has the following sensible communication in the St. Louis *Medical and Surgical Journal:* "About a quarter of a century ago, when giving some good advice for a young practitioner to follow, the late Dr. B. F. Edwards of St. Louis, Mo., whose action in the measurement of the action of remedies, truth in statement, and justice towards members of the profession, made him a shining light in the early history of our State, among other things says: "Never make fun of an old woman's remedy, for not only will you give offence and thereby injure your practice to the extent of her influence, but you may throw away what would have proved upon trial, a valuable adjunct in your practice? He then cited this case to illustrate the importance of his injunction: "In 1839, while practicing in Madison County, Ill., I was induced by representations of an old woman to make the trial, in dysntery and diarrhœa, of a table spoonful dose of *pure cider vinegar*, with the addition of sufficient salt to be noticeable, and it acted so charmingly that I never used anything else." He was prescribing it in 1870, making it a period of forty years.—*Medical and Surgical Reporter.*

### KINDNESS TO HORSES.

A Bath correspondent of the Boston Globe protests against putting a frosty bit into a horses mouth. He says, however, that rubber bits are unsafe, as they are liable to break, and always at the worst possible moment. A leather-covered bit is nasty. If one does not think so let him chew a piece of leather. A gill of hot water poured over the iron bit removes the frost and makes it warm and confortable. There is another thing fully as important, and that is the practice of giving horses and stock ice cold water. When the ther mometer registers zero, or even below freezing, and a pail or two of icy water is put into the animal's stomach, particualarly when he is to stand any length of time, it chills not only the stomach but the whole system, and an extra quantity of food is absolutely necessary to keep up the circulation. If to a pail of well or cold cistern water a quart of boiling water be added, the flnid passes into the animal's stomach at a blood-warm temperature, and no harm, but much good, results. The condition in which your horse comes out in the spring after the practice of this rule is ample evidence of its efficiency.

